# Prognostic Value of STAS, Lymph Node Metastasis, and VPI in NSCLC ≤ 4 cm Treated with Lobectomy

**DOI:** 10.3390/jcm15010233

**Published:** 2025-12-28

**Authors:** Esra Zeynelgil, Abdülkadir Koçanoğlu, Ata Türker Arıkök, Serdar Karakaya, Engin Eren Kavak, Tülay Eren

**Affiliations:** 1 Department of Medical Oncology, Ankara Etlik City Hospital, Ankara 06170, Turkey; engineren2000@yahoo.com (E.E.K.); tulayeren78@gmail.com (T.E.); 2 Department of Medical Oncology, Ankara Atatürk Sanatorium Training and Research Hospital, Ankara 06290, Turkey; kadirkocanoglu@hotmail.com (A.K.); drserdarkarakaya@gmail.com (S.K.); 3 Department of Pathology, Ankara Etlik City Hospital, Ankara 06170, Turkey; atalina2005@yahoo.com

**Keywords:** non-small cell lung cancer (NSCLC), spread through air spaces (STAS), disease-free survival (DFS), lobectomy, tumor ≤ 4 cm, visceral pleural invasion (VPI), lymphovascular invasion (LVI), lymph node metastasis, prognostic factors

## Abstract

**Background/Objectives:** This study aimed to evaluate the prognostic effects of tumor spread through air spaces (STAS) and other clinical and pathological risk factors on disease-free survival (DFS) in patients with non-small cell lung cancer (NSCLC) who underwent curative lobectomy and had tumors measuring 4 cm or less. **Methods**: NSCLC patients who underwent surgery between March 2015 and May 2024 and had at least 12 months of follow-up were retrospectively analyzed. Patients with tumors measuring 4 cm or less who underwent R0 resection, lobectomy, and STAS assessment on intraoperative frozen sections were included in the study. Clinicopathological features of all patients were restaged according to the 9th edition of the TNM staging system. The Kaplan–Meier method, log-rank test, and univariate Cox regression analysis were used to determine the factors affecting DFS. **Results:** 88 patients were included in the study. The median age of the patients was 61 years, 77.3% were male, and 72.7% had adenocarcinoma histology. According to TNM 9, 23.9% of the cases were staged T1b, 18.2% T1c, and 58.0% T2a. STAS positivity was detected in 45 patients (51.1%). The rates of lymphovascular invasion (LVI) (40.0% vs. 18.6%; *p* = 0.028) and visceral pleural invasion (VPI) (57.8% vs. 27.9%; *p* = 0.005) were significantly higher in the STAS-positive group than in the STAS-negative group. Recurrence was observed in a total of 31 patients (35.2%) during a median follow-up period of 68.1 months. In Kaplan–Meier analysis, the median DFS was not reached for the entire cohort. The estimated median DFS in STAS-positive patients was 52.7 months, while the median was not reached in the STAS-negative group (*p* = 0.001). The median DFS was 52.3 months in those with lymph node positivity, while the median was not reached in those with lymph node negativity (*p* = 0.031). According to TNM 9, the difference in DFS between stage IA/IB and stage IIAB groups was not statistically significant (*p* = 0.080). In univariate Cox analysis, STAS positivity (HR = 3.79; 95% CI: 1.69–8.51; *p* = 0.001), lymph node positivity (HR = 2.58; 95% CI: 1.05–6.31; *p* = 0.038) and VPI (HR = 2.28; 95% CI: 1.07–4.86; *p* = 0.032) were found to be significant prognostic factors adversely affecting DFS. Age, gender, histological type, tumor location, T stage, LVI, perineural invasion (PNI), and adjuvant chemotherapy had no significant effect on DFS. **Conclusions:** STAS is a strong negative prognostic indicator for recurrence in patients with operated NSCLC with tumor size ≤ 4 cm. It is believed that STAS should be integrated into risk-based staging and adjuvant treatment decision-making processes in early-stage NSCLC, particularly when evaluated in conjunction with VPI and lymph node positivity.

## 1. Introduction

Lung cancer is the second most common cancer in both men and women, yet it is the leading cause of cancer-related death [1]. Non-small cell lung cancer (NSCLC) accounts for approximately 85% of all lung cancer cases [2]. Surgical resection is the standard treatment for early-stage (stage I–II) NSCLC. Although curative treatment is possible at this stage, recurrent disease may occur in some of these patients. Comprehensive data on the relationship between stage and prognosis were obtained from a series used to validate the ninth edition of the Tumor, Lymph Node, and Metastasis Classification (TNM) system. Five-year survival rates were reported as 82% for stage IA, 69% for stage IB, 62% for stage IIA, and 54% for stage IIB, respectively [3].

Adjuvant chemotherapy modality for patients with operated lung cancer has become standard practice with the LACE meta-analysis. In a pooled analysis of 4584 patients, adjuvant chemotherapy contributed 5.4% to overall survival and 16% to the risk of recurrence [4]. However, meta-analysis findings revealed a trend toward a negative survival in stage IA patients receiving adjuvant chemotherapy [4]. Subsequent retrospective data have reported controversial findings regarding the benefit of adjuvant chemotherapy in high-risk Stage 1A patients. A retrospective study conducted in Japan reported that adjuvant chemotherapy in 641 stage IA patients with high-risk factors (e.g., invasive component >2 cm, visceral pleural invasion, lymphatic or vascular invasion) increased the five-year recurrence-free survival rate from 74% to 81% and the overall survival rate from 82% to 93% [5]. Therefore, identifying the high-risk patient population in whom adjuvant chemotherapy or adjuvant immunotherapy modalities should be administered is crucial in early-stage lung cancer [6].

The role of adjuvant chemotherapy in patients with resected stage IB lung cancer remains controversial. Historically, adjuvant chemotherapy has been considered primarily for tumors ≥4 cm in size. However, because previous TNM staging systems did not adequately incorporate additional risk factors such as visceral pleural invasion, the stage classification of patients with tumors smaller than 5 cm and concomitant visceral pleural invasion remained unclear [7]. A clear distinction was not made between T2a and T2b subtypes, and the stage assigned to tumors with risk factors such as visceral pleural invasion was unclear. The updated TNM 9 classification addressed some of this uncertainty [8]. All patients with tumors measuring ≤4 cm and visceral pleural invasion are now classified as Stage 1B (T2AN0), and tumors measuring 4–5 cm are classified as Stage 2 (T2BN0). This change demonstrates that the TNM 9 staging system adopts a more detailed, prognostic classification approach based on risk factors. This staging has highlighted the need to refine risk factors for tumors measuring 4 cm or less and define the at-risk population.

Spread-Through Air Spaces (STAS) is a histopathological finding in lung cancer pathology that describes the dissemination of tumor cells from the main mass throughout the alveolar air spaces [9]. According to the World Health Organization (WHO) definition, STAS appears as “micropapillary cell clusters, solid cell islands, or individual tumor cells spreading within the air spaces beyond the borders of the main tumor mass” [10]. Recent studies suggest a possible association between STAS positivity and poor prognosis [11,12]. However, the number of studies evaluating the prognostic impact of STAS in patients with tumors smaller than 4 cm is quite limited, and most of the available data are based on subgroup analyses. Therefore, STAS is not yet considered a definitive high-risk factor in guidelines [13,14]. Similarly, while visceral pleural invasion alone has an impact on staging in tumors 4 cm or less in TNM 9 staging, there have been suggestions to include STAS as a histological descriptor for the Ninth Edition of the TNM Classification of Lung Cancer [3,15].

In this study, we aimed to investigate the prognostic significance of STAS, other risk factors, and applied treatment modalities on recurrence in patients with surgically resected non-small cell lung cancer (NSCLC) with tumors measuring ≤4 cm.

## 2. Materials and Methods

### 2.1. Patient Characteristics and Data Collection

**Figure 1:**
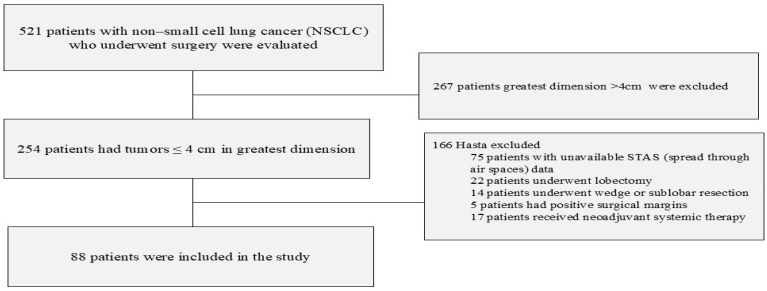
Flowchart of patient selection demonstrating inclusion and exclusion criteria.

This study was conducted by retrospectively reviewing the data of NSCLC patients who underwent surgery at Etlik City Hospital (formerly Dışkapı Training and Research Hospital, relocated) between March 2015 and May 2024 and had a minimum 12-month postoperative follow-up. The data cutoff was 1 May 2025. Inclusion criteria included having a tumor size of 4 cm or less, being 18 years of age or older, having undergone lobectomy, having negative surgical margins, having a definitive histological diagnosis of non-small cell lung cancer, and reporting STAS in the pathology report during frozen section examination. Exclusion criteria included having undergone R1-R2 resection, having received neoadjuvant chemotherapy, having received adjuvant immunotherapy (pembrolizumab or atezolizumab) after chemotherapy, patients with undetermined histological type, having secondary malignancies, and not having undergone wedge resection, sublobar resection, or pneumectomy (Figure 1).

Adjuvant chemotherapy was administered based on pathological stage, presence of high-risk features, and multidisciplinary tumor board recommendations. Chemotherapy regimens consisted of platinum-based doublets, including pemetrexed, gemcitabine, or vinorelbine, administered for four cycles when indicated. Patients were followed according to institutional surveillance protocols, including regular outpatient visits and radiological imaging. Disease-free survival (DFS) was defined as the interval from the date of surgery to the date of documented disease recurrence or last follow-up in patients without recurrence.

### 2.2. Pathological Assessment

Staging was performed retrospectively using the American College of Surgeons TNM Staging System, Ninth Edition [15]. All patients underwent anatomical lobectomy with curative intent. Systematic hilar and mediastinal lymph node sampling or dissection was performed according to institutional standards and surgeon preference. Lymph node status was determined based on pathological examination and classified as negative (N0) or positive (N1).

STAS was assessed intraoperatively in frozen surgical specimens. Under the microscope, STAS positivity was defined as the observation of free-floating tumor cells within normal alveolar structures, away from the primary tumor margins [16]. Patients suspected of having tumors disrupted by mechanical or knife action were reported as STAS negative.

Lymphovascular invasion (LVI) was defined as the presence of tumor cells within endothelial-lined lymphatic or vascular spaces in intratumoral or peritumoral areas and was evaluated on routine hematoxylin and eosin (H&E)–stained sections; only unequivocal intraluminal tumor cell clusters with identifiable endothelial lining were considered positive, while retraction artifacts were excluded. Perineural invasion (PNI) was defined as tumor cell infiltration within, around, or tracking along a nerve sheath and was recorded as present only when a direct tumor–nerve interface was observed on H&E sections. Visceral pleural invasion (VPI) was assessed in accordance with IASLC and AJCC criteria and defined as tumor invasion beyond the elastic layer of the visceral pleura; evaluation was primarily based on microscopic examination of H&E-stained sections, and VPI status was recorded as present or absent for pathological staging purposes according to the TNM 9th Edition.

### 2.3. Statistical Analysis

All statistical analyses used SPSS 24.0 (SPSS Inc., Chicago, IL, USA), R software (RStudio, version 2025.09.1+401; R Foundation for Statistical Computing, Vienna, Austria), and Microsoft^®^ Excel^®^ 2019 (32-bit). The data obtained in the study were summarized with descriptive statistics. Mean, median, and percentage values were calculated for continuous variables, and frequencies and ratios were calculated for categorical variables. Cross-tables were created to evaluate the relationship between categorical variables and STAS, and the chi-square test was applied to these tables. In cases where the assumptions of the chi-square test were not met, especially in 2 × 2 tables where the expected frequencies in the cells were below 5, Fisher’s Exact Test was used. Disease-free Survival (DFS) was calculated as the time until recurrence of disease after surgery and as the date of last follow-up in patients without recurrence. The Kaplan–Meier method was used to calculate the estimated median DFS, and *p* values were obtained using the Log-Rank test to compare variables, and survival graphs were plotted. Univariate hazard ratios for overall survival were estimated using Cox proportional hazards regression. Multivariate analysis was conducted employing a forward stepwise selection approach based on the likelihood ratio test. Variables with a *p*-value < 0.05 were considered statistically significant and were retained in the model according to predefined inclusion criteria. The final step of the stepwise procedure was reported as the definitive multivariate model. In all statistical analyses, a *p* value of <0.05 was considered statistically significant. The Type I error level was set at 5%. Given the retrospective nature of the study, a formal a priori sample size calculation was not performed. However, a post hoc power consideration was conducted based on the observed effect size. With 88 patients and 31 recurrence events, the study had adequate statistical power (>80%) to detect the observed hazard ratio for STAS (HR ≈ 3.8) at a two-sided alpha level of 0.05.

## 3. Results

A total of 88 patients were included in the study. The median age was 61 years (range: 18–78), and the median follow-up duration was 68.1 months. The mean follow-up duration was 67.5 ± 29.6 months. Of all patients, 77.3% (*n* = 68) were male, and 72.7% (*n* = 64) had adenocarcinoma histology. According to the 9th edition of the TNM classification, 23.9% were stage T1b (*n* = 21), 18.2% (*n* = 16) were T1c, and 58.0% (*n* = 51) were T2a. LVI was identified in 29.5% (*n* = 26), and PNI in 13.6% (*n* = 12). STAS was present in 51.1% (*n* = 45), and visceral pleural invasion in 43.2% (*n* = 38). In total, 42.0% of the patients (*n* = 37) received adjuvant chemotherapy. Additional variables are presented in Table 1.

The clinicopathological features of STAS-positive (*n* = 45) and STAS-negative (*n* = 43) patients were compared. No statistically significant differences were detected between the two groups in terms of age, sex, histological type, tumor location, tumor T stage, lymph node status, overall stage, or adjuvant chemotherapy (all *p* > 0.05). LVI was observed in 40.0 (*n* = 18) of STAS-positive cases and in 18.6% (*n* = 8) of STAS-negative cases (*p* = 0.028). Visceral pleural invasion was detected in 57.8% (*n* = 26) of STAS-positive patients and in 27.9% (*n* = 12) of STAS-negative patients (*p* = 0.005). Perineural invasion was more frequent in STAS-positive cases (20.0%, *n* = 9), and the statistical significance approached but did not reach the threshold (*p* = 0.051) (Table 1).

**Figure 2:**
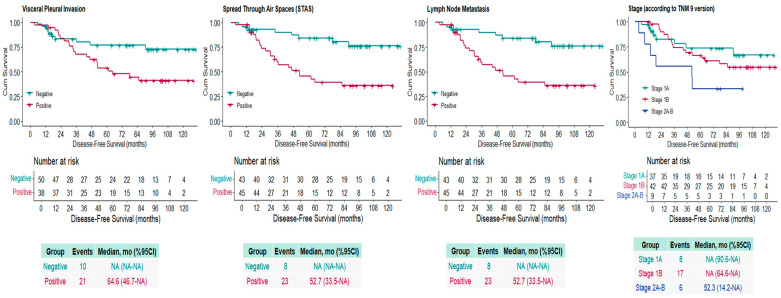
Kaplan–Meier Disease-Free Survival (DFS) chart for stage, visceral pleural invasion (VPI), tumor spread through air spaces (STAS), and lymph node metastasis. NA: Not available.

Disease recurrence occurred in 31 patients (35.2%). In Kaplan–Meier analysis, the median DFS for the entire cohort was not reached. The estimated median DFS was 52.7 months for the STAS-positive group, while the median was not reached for the STAS-negative group (log-rank *p* = 0.001). The estimated median DFS was 52.3 months for patients with lymph node metastasis, while the median was not reached for those without metastasis (log-rank *p* = 0.031). The median DFS was not reached for stage IA and IB patients. For stage IIA–IIB cases (T1 or T2 with N1 involvement), DFS was 52.3 months (log-rank *p* = 0.080). The estimated DFS was 64.6 months in patients with visceral pleural invasion, while the median was not reached in those without invasion (log-rank *p* = 0.028) (Figure 2).

**Figure 3:**
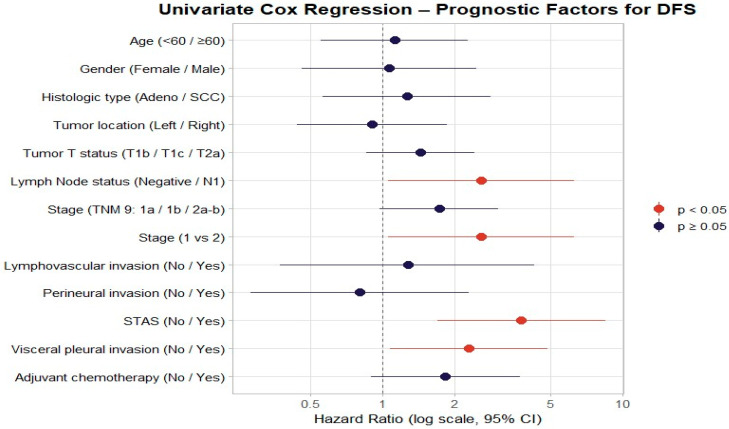
Forest plot for disease-free survival, showing hazard ratios and 95% confidence intervals for subgroup analyses.

In the univariate Cox regression analysis for DFS, lymph node positivity (HR = 2.58, 95% CI: 1.05–6.31, *p* = 0.038), stage II disease (HR = 2.58, 95% CI: 1.05–6.31, *p* = 0.038), STAS positivity (HR = 3.79, 95% CI: 1.69–8.51, *p* = 0.001), and visceral pleural invasion (HR = 2.28, 95% CI: 1.07–4.86, *p* = 0.032) were identified as poor prognostic indicators. Age (HR = 1.12, 95% CI: 0.55–2.27, *p* = 0.757), sex (HR = 1.06, 95% CI: 0.46–2.46, *p* = 0.894), histological type (HR = 1.26, 95% CI: 0.56–2.82, *p* = 0.576), tumor location (HR = 0.90, 95% CI: 0.44–1.85, *p* = 0.780), T stage (HR = 1.43, 95% CI: 0.85–2.41, *p* = 0.175), lymphovascular invasion (HR = 1.35, 95% CI: 0.63–2.86, *p* = 0.442), perineural invasion (HR = 0.80, 95% CI: 0.28–2.29, *p* = 0.678), and adjuvant chemotherapy (HR = 1.82, 95% CI: 0.89–3.76, *p* = 0.104) showed no significant association with DFS (Table 2) (Figure 3).

In multivariate Cox regression analysis, parameters with statistical significance in univariate analysis (lymph node metastasis-stage, visceral pleural invasion, and STAS) were included in the analysis using the forward-LR stepwise method. STAS (HR: 4.43 95% CI: 1.94–10.14, *p* < 0.001) and lymph node metastasis-stage (HR: 3.67 95% CI: 1.47–9.21, *p* = 0.006) showed a statistically significant model feature for DFS.

A subgroup analysis was performed on STAS-positive patients (*n* = 45). In univariate Cox regression analysis for DFS in STAS-positive patients, no statistically significant relationship was found between age (*p* = 0.108), gender (*p* = 0.244), histological subtype (*p* = 0.192), tumor localization (*p* = 0.660), primary tumor stage (*p* = 0.242), and lymphovascular invasion (*p* = 0.953). Although pathological stage and perineural invasion showed a borderline significant relationship with DFS, these relationships did not reach statistical significance (*p* = 0.056 and *p* = 0.059, respectively). Patients with lymph node involvement (HR = 4.57; 95% CI: 1.52–13.82, *p* = 0.007), visceral pleural invasion (HR = 4.81; 95% CI: 1.42–16.30, *p* = 0.012), and those who received adjuvant chemotherapy (HR = 3.00; 95% CI: 1.18–7.66, *p* = 0.021) had a poor prognosis. The three statistically significant parameters were removed using multivariate Cox regression analysis and a forward-LR stepwise model. In multivariate analysis, lymph node involvement (HR = 3.12; 95% CI: 1.02–9.58, *p* = 0.047) and visceral pleural invasion (HR = 4.20; 95% CI: 1.21–14.51, *p* = 0.023) were found to be significantly associated with disease-free survival (Supplement Table S1). In subgroup analysis of patients without lymph node metastasis (*n* = 79), the presence of STAS (HR: 3.94, 95% CI: 1.57–9.90, *p* = 0.004) and visceral pleural invasion (HR: 2.33, 95% CI: 1.01–5.42, *p* = 0.049) were prognostic features for DFS. In multivariate analysis, the presence of STAS (HR: 3.39, 95% CI: 1.30–8.82, *p* = 0.012) maintained statistical significance. (Supplement Table S2, Supplement Figure S1)

## 4. Discussion

In this study, 88 patients with NSCLC 4 cm or less who underwent lobectomy were analyzed. Lymph node metastasis (HR: 2.58, *p* = 0.038), visceral pleural invasion (HR: 2.28, *p* = 0.032), and STAS (HR: 3.79, *p* = 0.001) were identified as poor prognostic indicators. Patients classified as stage II due to the presence of lymph node metastasis were statistically shown to have a poorer prognostic value compared to stage 1 patients in terms of DFS (HR: 2.58, 0.038). However, although stages 1A, 1B, and 2AB differed in terms of prognosis, they did not reach statistical significance (HR: 1.72, *p* = 0.064).

The pathophysiology and formation mechanism of STAS have not yet been fully clarified. Some hypotheses suggest that tumor cells may passively disseminate within the lung parenchyma via the airways, while other views suggest that these cell clusters spread into the alveolar spaces through active invasion [17]. However, whether STAS is an in vivo effect or an artifact of tumor excision with a knife remains controversial [18]. STAS, commonly examined intraoperatively by frozen section, can be detected with positivity between 15% and 62% in operated NSCLC patients [19,20,21]. In our study, STAS positivity was found in 51.1% of patients with tumors 4 cm or smaller, which is consistent with the literature.

Studies have investigated the relationship between STAS and prognosis in patients with NSCLC who underwent surgery. Yanagawa et al. found that the incidence of STAS in surgical resection of squamous cell carcinoma was 19.1% and that stage I lung squamous cell carcinoma was associated with a 5-year DFS, but there was no statistical difference between stages II and III [22]. Yang et al. studied 242 patients with stage I lung adenocarcinoma and concluded that STAS was an independent prognostic risk factor in patients with radical lobectomy who had a tumor diameter of 2 to 4 cm, but was not prognostic in tumors ≤2 cm [23]. Shiono et al. reported that STAS was significantly associated with lymphatic vessel and pleural invasion in 318 stage I lung adenocarcinoma cases, and that STAS-positive cases had significantly lower five-year survival rates (STAS positive = 54.4%, STAS negative = 87.7%, *p* < 0.01) [24]. Yi et al. demonstrated in a cohort of 109 patients that the presence of STAS may be a significant predictor of recurrence in patients with early-stage aggressive lung adenocarcinoma treated with lobectomy (HR = 5.9, *p* = 0.031) [25]. In our study, a positive association was found between LVI and visceral pleural invasion and STAS positivity (*p* = 0.028, *p* = 0.032, respectively), and it was concluded that STAS is a poor prognostic indicator for recurrent disease in positive patients (HR: 3.79, 95% CI: 1.69–8.51, *p* = 0.001). Adjuvant chemotherapy after curative resection is not routinely recommended for patients with NSCLC <4 cm, but it is concluded that tumors <4 cm should be evaluated for adjuvant chemotherapy with stage 1b (T2AN0M0) along with risk factor staging according to TNM 9, and risk factor classification such as visceral pleural invasion [15]. Du et al.’s meta-analysis of 2899 patients supports the need for STAS-based staging [26].

In addition to its value as a prognostic marker, emerging evidence indicates that STAS may also play a role in shaping surgical decision-making. Studies by Kadota et al. demonstrated that STAS serves as an independent predictor of recurrence in early-stage lung cancer treated with sublobar resection, a relationship that was not seen in patients undergoing lobectomy [27]. Similar results were reported by Eguchi et al. in a cohort of 1497 patients and by Shiono et al. in a cohort of 514 patients [28]. These findings collectively suggest that, even with negative surgical margins, sublobar resection may compromise outcomes in STAS-positive patients [29].

Conflicting data exist concerning the prognostic impact of STAS. Uruga et al. and Toyokawa et al. reported that STAS was not an independent prognostic factor after adjustment for tumor size, histologic subtype, and extent of resection, indicating that its prognostic effect may be context-dependent [19,30]. Similarly, Warth et al. highlighted methodological limitations in STAS assessment, and a subsequent meta-analysis by Chen et al. revealed substantial heterogeneity across studies, underscoring the lack of standardization and the need for prospective validation [20,31]. In this regard, STAS may represent biological information complementary to, but not currently integrated within, size-based TNM-9 T descriptors, underscoring the need for prospective studies to define its role alongside revised T2a/T2b criteria. Furthermore, routine evaluation of STAS, while requiring an increase in pathological assessment time and expertise, may provide a significant cost–benefit advantage by identifying high-risk patients who would benefit from more extensive surgery or adjuvant treatment to prevent expensive recurrence-related outcomes.

There were some limitations to our study. Our study was based on retrospective data. Furthermore, the inability to determine true STAS positive and true STAS negative rates due to the lack of a gold standard method was another limitation. The lack of a gold standard method for STAS and the failure to detect STAS in 15% of cases due to artifacts represent a significant measurement problem [32]. Moreover, since our cohort consists exclusively of patients who underwent lobectomy, these results cannot be directly generalized to other surgical approaches. The lack of statistical significance with adjuvant chemotherapy (*p* = 0.104) may be due to the reduced risk of recurrence in most high-risk groups receiving adjuvant chemotherapy. In addition to these limitations, the presence of STAS was determined based on frozen section evaluation, which has lower diagnostic accuracy compared with permanent histopathological sections. Therefore, the assessment of STAS in this study may be subject to misclassification bias, and further validation using conventional sectioning methods is required. Finally, the relatively small sample size, particularly in certain subgroups such as patients with lymph node metastasis, limits the statistical power and generalizability of subgroup analyses. Although the study was sufficiently powered to detect the main effect of STAS on disease-free survival, these subgroup findings should be considered exploratory rather than definitive.

## 5. Conclusions

In conclusion, our study demonstrates that STAS, together with lymph node metastasis and visceral pleural invasion, is a strong adverse prognostic factor in patients with resected NSCLC ≤ 4 cm (T1–T2a), a group in which the benefit of adjuvant chemotherapy remains uncertain. We concluded that STAS could be added to the risk-based staging system in this patient population, in addition to visceral pleural invasion. Although our study found STAS to be a strong prognostic marker, these findings need to be validated in larger populations. For example, the International Association for the Study of Lung Cancer (IASLC) study of 4061 pathological Stage I NSCLC patients, which provided recommendations for the 9th edition of the TNM classification, suggested the inclusion of STAS as a histological descriptor in the system [15]. While our findings are consistent with these large-scale validation studies, more prospective and multicenter data are needed for the full integration of STAS into the staging system. Future studies require innovations in adjuvant treatment strategies based on genomic profiles or adjuvant chemotherapy with other risk factors, along with STAS, for tumors 4 cm or less. Furthermore, tests capable of detecting STAS with high specificity and sensitivity, both preoperatively and intraoperatively, need to be developed.

## Figures and Tables

**Table 1:** Patient characteristics and relationship between variables and STAS.

Variable	Category	*n*	%	STAS Negative *n* = 43	STAS Positive *n* = 45	*p* *
Age	<60	35	39.8	17 (39.5%)	18 (40.0%)	0.964
	≥60	53	60.2	26 (60.5%)	27 (60.0%)	
Gender	Female	20	22.7	6 (14.0%)	14 (31.1%)	0.055
	Male	68	77.3	37 (86.0%)	31 (68.9%)	
Histologic type	Adenocarcinoma	64	72.7	31 (72.1%)	33 (73.3%)	0.896
	Squamous cell	24	27.3	12 (27.9%)	12 (26.7%)	
Tumor location	Left	38	43.2	16 (37.2%)	22 (48.9%)	0.269
	Right	50	56.8	27 (62.8%)	23 (51.1%)	
Tumor T	T1b	21	23.9	12 (27.9%)	9 (20.0%)	0.226
	T1c	16	18.2	10 (23.3%)	6 (13.3%)	
	T2a	51	58.0	21 (48.8%)	30 (66.7%)	
Node status	Negative	79	89.8	38 (88.4%)	41 (91.1%)	0.672
	Positive	9	10.2	5 (11.6%)	4 (8.9%)	
Stage	Stage 1A	37	42.0	22 (51.2%)	15 (33.3%)	0.152
	Stage 1B	42	47.7	16 (37.2%)	26 (57.8%)	
	Stage 2A-B	9	10.2	5 (11.6%)	4 (8.9%)	
Lymphovascular invasion	No	62	70.5	35 (81.4%)	27(60.0%)	**0.028**
	Yes	26	29.5	8(18.6%)	18 (40.0%)	
Perineural invasion	No	76	86.4	34 (79.1%)	42 (93.3%)	0.051
	Yes	12	13.6	9 (20.9%)	3 (6.7%)	
Spread through air spaces	No	43	48.9	N.A.	N.A.	N.A.
	Yes	45	51.1	N.A.	N.A.	
Visceral pleural invasion	No	50	56.8	31 (72.1%)	19 (42.2%)	**0.005**
	Yes	38	43.2	12 (27.9%)	26 (57.8%)	
Adjuvant chemotherapy	No	51	58.0	28 (65.1%)	23 (51.1%)	0.183
	Yes	37	42.0	15 (34.9%)	22 (48.9%)	

* Statistically significant *p* values are marked in bold. N.A.: Not Applicable.

**Table 2:** Univariate Cox-Regression analysis of prognostic factors affecting disease-free survival (DFS) in patients with operated lung cancer ≤4 cm.

		Univariate Analysis		Multivariate Analysis	
Variable	Category	HR (95% CI)	*p* *		*p* **
Age	<60/≥60	1.12 (0.55–2.27)	0.757		
Gender	Female/Male	1.06 (0.46–2.46)	0.894		
Histologic type	Adenocarcinoma/SCC	1.26 (0.56–2.82)	0.576		
Tumour location	Left/Right	0.90 (0.44–1.85)	0.780		
Primary tumour	T1b/T1c/T2a	1.43 (0.85–2.41)	0.175		
Lymph Node status	Negative/N1	2.58 (1.05–6.31)	**0.038**	3.67 (1.47–9.21)	**0.006**
Stage	1a/1b/2a-b	1.72 (0.97–3.04)	0.064		
Stage (1 vs. 2)	1/2	2.58 (1.05–6.31)	**0.038**		
Lymphovascular invasion	No/Yes	1.35 (0.63–2.86)	0.442		
Perineural invasion	No/Yes	0.80 (0.28–2.29)	0.678		
STAS	No/Yes	3.79 (1.69–8.51)	**0.001**	4.43 (1.94–10.14)	**<0.001**
Visceral pleural invasion	No/Yes	2.28 (1.07–4.86)	**0.032**		
Adjuvant chemotherapy	No/Yes	1.82 (0.89–3.76)	0.104		

* Statistically significant *p* values are marked in bold **. In multivariate Cox regression analysis, parameters with statistical significance in univariate analysis (lymph node metastasis-stage, visceral pleural invasion, and STAS) were included in the analysis using the forward-LR stepwise method.

## Data Availability

The datasets generated and analyzed during the current study are available from the corresponding author upon reasonable request.

## Supplementary Materials

The following supporting information can be downloaded at: https://www.mdpi.com/article/10.3390/jcm15010233/s1, Table S1: Univariate and Multivariate Analyses of Factors Affecting Disease-Free Survival in STAS-Positive Patients (*n* = 45); Table S2: Univariate and Multivariate Analyses of Factors Affecting Disease-Free Survival in the Lymph Node–Negative Subgroup (*n* = 79); Figure S1: Forest plot of the univariate analysis in lymph node metastasis–negative patients (*n* = 79), illustrating disease-free survival with hazard ratios and 95% confidence intervals for subgroup analyses.

## Abbreviations

The following abbreviations are used in this manuscript:

NSCLC	Non-Small Cell Lung Cancer
STAS	Spread Through Air Spaces
DFS	Disease-Free Survival
TNM	Tumor-Node-Metastasis
LVI	Lymphovascular Invasion
VPI	Visceral Pleural Invasion
PNI	Perineural Invasion
HR	Hazard Ratio
CI	Confidence Interval
R0	Microscopically Margin-Negative Resection

## References

[1] Siegel R.L., Kratzer T.B., Giaquinto A.N., Sung H., Jemal A. (2025). Cancer statistics, 2025. CA Cancer J. Clin..

[2] Sung H., Ferlay J., Siegel R.L., Laversanne M., Soerjomataram I., Jemal A., Bray F. (2021). Global cancer statistics 2020: GLOBOCAN estimates of incidence and mortality worldwide for 36 cancers in 185 countries. CA Cancer J. Clin..

[3] Amin M.B., Edge S.B. (2024). AJCC Cancer Staging System.

[4] Pignon J.-P., Tribodet H., Scagliotti G.V., Douillard J.-Y., A Shepherd F., Stephens R.J., Dunant A., Torri V., Rosell R., Seymour L. (2008). Lung adjuvant cisplatin evaluation: A pooled analysis by the LACE Collaborative Group. J. Clin. Oncol..

[5] Tsutani Y., Imai K., Ito H., Mimae T., Miyata Y., Ikeda N., Nakayama H., Okada M. (2019). Adjuvant chemotherapy for pathological stage I non-small cell lung cancer with high-risk factors for recurrence: A multicenter study. J. Clin. Oncol..

[6] Felip E., Altorki N., Zhou C., Csőszi T., Vynnychenko I., Goloborodko O., Luft A., Akopov A., Martinez-Marti A., Kenmotsu H. (2021). Adjuvant atezolizumab after adjuvant chemotherapy in resected stage IB–IIIA non-small-cell lung cancer (IMpower010): A randomised, multicentre, open-label, phase 3 trial. Lancet.

[7] Lim W., Ridge C.A., Nicholson A.G., Mirsadraee S. (2018). The 8th lung cancer TNM classification and clinical staging system: Review of the changes and clinical implications. Quant. Imaging Med. Surg..

[8] Rami-Porta R., Nishimura K.K., Giroux D.J., Detterbeck F., Cardillo G., Edwards J.G., Fong K.M., Giuliani M., Huang J., Kernstine K.H. (2024). The IASLC Lung Cancer Staging Project: Proposals for Revision of the TNM Stage Groups in the Forthcoming (Ninth) Edition of the TNM Classification for Lung Cancer. J. Thorac. Oncol..

[9] Warth A. (2017). Spread through air spaces (STAS): A comprehensive update. Transl. Lung Cancer Res..

[10] Travis W.D., Brambilla E., Nicholson A.G., Yatabe Y., Austin J.H.M., Beasley M.B., Chirieac L.R., Dacic S., Duhig E., Flieder D.B. (2015). The 2015 World Health Organization classification of lung tumors: Impact of genetic, clinical and radiologic advances since the 2004 classification. J. Thorac. Oncol..

[11] Jia M., Yu S., Gao H., Sun P.-L. (2020). Spread through air spaces (STAS) in lung cancer: A multiple-perspective and update review. Cancer Manag. Res..

[12] Jiang M.-Q., Qian L.-Q., Shen Y.-J., Fu Y.-Y., Feng W., Ding Z.-P., Han Y.-C., Fu X.-L. (2024). Who benefit from adjuvant chemotherapy in stage I lung adenocarcinoma? A multi-dimensional model for candidate selection. Neoplasia.

[13] Wood D.E., Kazerooni E.A., Aberle D.R., Argento C., Baines J., Boer B., Brown L.M., Donington J., Eapen G.A., Ferguson J.S. (2025). NCCN Guidelines^®^ Insights: Lung Cancer Screening, Version 1.2025: Featured Updates to the NCCN Guidelines. J. Natl. Compr. Cancer Netw..

[14] Remon J., Soria J.-C., Peters S. (2021). Early and locally advanced non-small-cell lung cancer: An update of the ESMO Clinical Practice Guidelines focusing on diagnosis, staging, systemic and local therapy. Ann. Oncol..

[15] Travis W.D., Eisele M., Nishimura K.K., Aly R.G., Bertoglio P., Chou T.-Y., Detterbeck F.C., Donnington J., Fang W., Joubert P. (2024). The international association for the study of lung cancer (IASLC) staging project for lung cancer: Recommendation to introduce spread through air spaces as a histologic descriptor in the ninth edition of the TNM classification of lung cancer. Analysis of 4061 pathologic stage I NSCLC. J. Thorac. Oncol..

[16] Ma K., Zhan C., Wang S., Shi Y., Jiang W., Wang Q. (2019). Spread through air spaces (STAS): A new pathologic morphology in lung cancer. Clin. Lung Cancer.

[17] Warth A., Muley T., Harms A., Hoffmann H., Dienemann H., Schirmacher P., Weichert W. (2016). Clinical relevance of different papillary growth patterns of pulmonary adenocarcinoma. Am. J. Surg. Pathol..

[18] Thunnissen E., Blaauwgeers H.J.L.G., de Cuba E.M.V., Yick C.Y., Flieder D.B. (2016). Ex vivo artifacts and histopathologic pitfalls in the lung. Arch. Pathol. Lab. Med..

[19] Toyokawa G., Yamada Y., Tagawa T., Kozuma Y., Matsubara T., Haratake N., Takamori S., Akamine T., Oda Y., Maehara Y. (2018). Significance of spread through air spaces in resected pathological stage I lung adenocarcinoma. Ann. Thorac. Surg..

[20] Warth A., Muley T., Kossakowski C.A., Goeppert B., Schirmacher P., Dienemann H., Weichert W. (2015). Prognostic impact of intra-alveolar tumor spread in pulmonary adenocarcinoma. Am. J. Surg. Pathol..

[21] Lee T.-H., Kuo C.-Y., Shen Y.-W., Kao S.-Y., Liu Y.-W., Lee J.-Y., Chuang C.-H., Lai W.-A., Wu C.-C., Lee M.-S. (2025). Prognostic significance of tumor spread through air spaces and lymphovascular invasion in stage I non-small cell lung cancer: Implications for adjuvant chemotherapy. World J. Surg. Oncol..

[22] Yanagawa N., Shiono S., Endo M., Ogata S. (2018). Tumor spread through air spaces is a useful predictor of recurrence and prognosis in stage I lung squamous cell carcinoma, but not in stage II and III. Lung Cancer.

[23] Yang L., Yang Y., Ma P., Zheng B., Liu W., Zhang Z., Ding N., Liu L., Mao Y., Lv N. (2018). Spread through air spaces predicts a worse survival in patients with stage I adenocarcinomas >2 cm after radical lobectomy. J. Thorac. Dis..

[24] Shiono S., Yanagawa N. (2016). Spread through air spaces is a predictive factor of recurrence and a prognostic factor in stage I lung adenocarcinoma. Interact. Cardiovasc. Thorac. Surg..

[25] Yi E., Lee J.H., Jung Y., Chung J.H., Lee Y., Lee S. (2021). Clinical implication of tumour spread through air spaces in pathological stage I lung adenocarcinoma treated with lobectomy. Interact. Cardiovasc. Thorac. Surg..

[26] Du H., Li X., Wang Y., Wang Q., Tao Y., Cui X., Wen Z., Yan S., Wu N. (2025). Benefit of adjuvant chemotherapy for resected stage I lung cancer with spread through air spaces: A systematic review and meta-analysis. Eur. J. Surg. Oncol..

[27] Kadota K., Kushida Y., Kagawa S., Ishikawa R., Ibuki E., Inoue K., Go T., Yokomise H., Ishii T., Kadowaki N. (2019). Limited resection is associated with a higher risk of locoregional recurrence than lobectomy in stage I lung adenocarcinoma with tumor spread through air spaces. Am. J. Surg. Pathol..

[28] Eguchi T., Kameda K., Lu S., Bott M.J., Tan K.S., Montecalvo J., Chang J.C., Rekhtman N., Jones D.R., Travis W.D. (2019). Lobectomy is associated with better outcomes than sublobar resection in spread through air spaces (STAS)-positive T1 lung adenocarcinoma: A propensity score–matched analysis. J. Thorac. Oncol..

[29] Ren Y., Xie H., Dai C., She Y., Su H., Xie D., Zheng H., Zhang L., Jiang G., Wu C. (2019). Prognostic impact of tumor spread through air spaces in sublobar resection for 1A lung adenocarcinoma patients. Ann. Surg. Oncol..

[30] Uruga H., Fujii T., Miyamoto A., Hisashi T. (2019). What did the first meta-analysis of tumor spread through air spaces (STAS) bring to light?. J. Thorac. Dis..

[31] Chen D., Mao Y., Wen J., She Y., Zhu E., Zhu F., Zhang Y., Fan M., Chen C., Chen Y. (2019). Tumor spread through air spaces in non-small cell lung cancer: A systematic review and meta-analysis. Ann. Thorac. Surg..

[32] Zhou F., Villalba J.A., Sayo T.M.S., Narula N., Pass H., Mino-Kenudson M., Moreira A.L. (2022). Assessment of the feasibility of frozen sections for the detection of spread through air spaces (STAS) in pulmonary adenocarcinoma. Mod. Pathol..

