# Supplementary material for: Prognostic Value of STAS, Lymph Node Metastasis, and VPI in NSCLC ≤ 4 cm Treated with Lobectomy
Source: J Clin Med. 2025 Dec 28;15(1):233. doi: 10.3390/jcm15010233 (PMC12786870; doi:10.3390/jcm15010233)
Supplement: Supplementary file 1 [file jcm-15-00233-s001.zip › jcm-4028018-supplementary.pdf]

Supplement Table S1. Univariate and Multivariate Analyses of Factors Affecting Disease-Free Survival in STAS-Positive Patients (n = 45)

		**Univariate analysis**		**Multivariate analysis**	
**Variable**	**Category**	**HR (95% CI)**	**p-value**	**HR (95% CI)**	**p-value**
Age	<60 / ≥60	2.03 (0.86–4.80)	0.108	-	-
Gender	Female / Male	1.74 (0.68–4.44)	0.244	-	-
Histologic type	Adenocarcinoma / SCC	1.82 (0.74–4.46)	0.192	-	-
Tumour location	Left / Right	1.21 (0.52–2.80)	0.660	-	-
Primary tumour	T1b / T1c / T2a	1.53 (0.75–3.14)	0.242	-	-
Lymph Node status	Negative / N1	4.57 (1.52–13.82)	**0.007**	3.12 (1.02–9.58)	**0.047**
Stage	1a / 1b / 2a-b	2.33 (0.98–5.51)	0.056	-	-
Lymphovascular invasion	No / Yes	1.03 (0.44–2.42)	0.953	-	-
Perineural invasion	No / Yes	3.28 (0.95–11.27)	0.059	-	-
Visceral pleural invasion	No / Yes	4.81 (1.42–16.30)	**0.012**	4.20 (1.21–14.51)	**0.023**
Adjuvant chemotherapy	No / Yes	3.00 (1.18–7.66)	**0.021**	-	-

Statistically significant p values are marked in bold

Supplement Table S2. Univariate and Multivariate Analyses of Factors Affecting Disease-Free Survival in the Lymph Node–Negative Subgroup (n = 79)

		Univariate analysis		Multivariate analysis*	
**Variable**	**Category**	**HR (95% CI)**	**p-value**	**HR (95% CI)**	**p-value**
Age	<60 / ≥60	1.02 (0.46–2.24)	0.960	–	–
Gender	Female / Male	1.01 (0.40–2.53)	0.981	–	–
Histologic type	Adenocarcinoma / SCC	1.20 (0.48–3.00)	0.704	–	–
Tumour location	Left / Right	0.89 (0.40–1.98)	0.774	–	–
Primary tumour	T1b / T1c / T2a	1.49 (0.88–2.52)	0.136	–	–
Stage	1a / 1b	1.30 (0.56–3.01)	0.543	–	–
Lymphovascular invasion	No / Yes	0.95 (0.36–2.53)	0.917	–	–
Perineural invasion	No / Yes	0.04 (0.00–10.10)	0.256	–	–
STAS	No / Yes	**3.94 (1.57–9.90)**	**0.004**	**3.39 (1.30–8.82)**	**0.012**
Visceral pleural invasion	No / Yes	**2.33 (1.01–5.42)**	**0.049**	1.65 (0.69–3.95)	0.263
Adjuvant chemotherapy	No / Yes	1.55 (0.71–3.42)	0.275	–	–

Statistically significant p values are marked in bold , *The enter method was used in the multivariate analysis

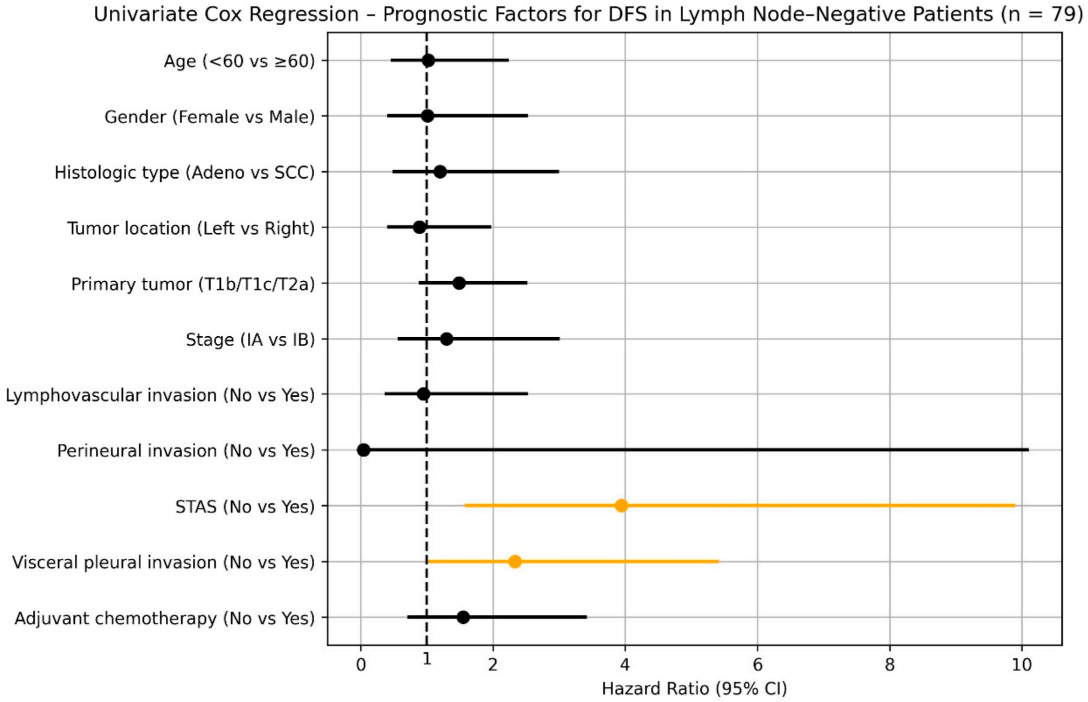

Supplement Figure S1. Forest plot of the univariate analysis in lymph node metastasis–negative patients (n=79), illustrating disease-free survival with hazard ratios and 95% confidence intervals for subgroup analyses
